# Rare Lipomatous Tumors with Osseous and/or Chondroid Differentiation in the Oral Cavity Report of Two Cases and Review of the Literature

**DOI:** 10.1155/2009/143460

**Published:** 2009-11-04

**Authors:** Kayo Kuyama, Sisilia Fusi Fifita, Masamichi Komiya, Yan Sun, Yoshiaki Akimoto, Hirotsugu Yamamoto

**Affiliations:** ^1^Department of Oral Pathology, Nihon University School of Dentistry at Matsudo, Chiba 271-8587, Japan; ^2^Department of Oral Surgery, Nihon University Itabashi Hospital, Tokyo 173-8610, Japan; ^3^Department of Oral Surgery, Nihon University School of Dentistry at Matsudo, Chiba 271-8587, Japan

## Abstract

The purpose of this study was to determine the clinicopathological and immunohistochemical features of lipoma/fibrolipoma with rare occasions as osseous and/or chondroid differentiation in the oral cavity. Two cases of the tumors, who presented with a painless, relatively hard mass on the oral mucosa, were studied. These were consisted of a well-circumscribed mass of fatty tissue with chondroid and significant fibrous component intermixed with the lobules of fat cells with chondroid and woven bone component, respectively. Immunohistochemical study revealed that peripheral spindle cells around chondroid tissue stained diffusely for S-100 *α* & *β* and Sox-9, though peripheral spindle cells around osteoid tissue only stained for RUNX-2. According to review of the literature, lipoma/fibrolipoma with osseous and/or chondroid differentiation was 18 cases. Also fibrolipoma with osseous and chondroid differentiation is the first to be reported here. These results indicated that the cartilage/bone is produced by differentiation of undifferentiated mesenchymal cells of stroma.

## 1. Introduction

Lipomas represent uncommon neoplasms of the oral cavity; only 1% to 5% of cases occur at this site [1]. Based on their histopathological features of conspicuous multiple components, lipomas can be divided into some subclasses [2, 3], and the most common is the fibrolipoma [1, 3, 4]. Few series of intraoral lipomas with osseous/chondroid differentiaion are seen in literature. Cases reporting on lipoma with osteo/chondroid differentiaion were only 16 cases as a result of retrieving literature from 1960 to 2008. Further, other names of this subclassification exist in literature; osteo/chondrolipoma, ossifying/osseous lipoma, lipoma with chondro/osseous metaplasia, and lipoma with cartilaginous/osseous change. The obscure etiology of osseous/chondroid differentiaion led to confusion of the name.

To clarify the etiology of osseous/chondroid differentiaion in lipoma, the authors report additional 2 cases of oral lipoma/fibrolipoma with osseous and/or chondroid differentiation and describe the clinical, histopathological, and immunohistological features of these. Review of the literature and clinicopathological analysis from the files of the Oral Pathology Department, Nihon University School of Dentistry at Matsudo from 1995 to 2007 of these cases were performed and compared.

## 2. Materials and Methods

### 2.1. Case Report

Two cases that had been diagnosed as lipoma with osteo/chondroid differentiation were retrieved from the files mentioned earlier. In these cases, the hematoxylin and eosin stained (H&E) slides were reviewed clinically and histopathologically.

### 2.2. Immunohistochemical Study

Sections of 2 cases were deparaffinized in xylene and dehydrated in Tris-buffered saline (pH 7.6). Primary antibodies against E29 (epithelial membrane antigen (EMA)), QBEnd 10 (CD34 Class II), Ki-S5 (Ki-67 Antigen), 5.8A (Myo D1) were purchased from a commercial source (Dako, Denmark), and sc-12488 (RUNX-2), sc-20095 (Sox-9), sc-71992 (S-100 *α* chain), sc-71993 (S-100 *β* chain), sc-55520 (FGF-1), and sc-57494 (VEGF) were purchased from a commercial source (Santa Cruz Biotechnology, Inc, USA). For detection of the antigen, the dextran polymer method (Chem Mate Envision kit, Dako, Denmark) was used. To improve detection, the deparaffinized sections were pretreated by microwave heating with citrate buffer (pH 6). The primary antibodies were generally used at a dilution of 1 : 50, and the incubation time was 1 hour at room temperature. Peroxidase activity was visualized using diaminobenzidine. Positive controls consisted of specimens of schwannoma for S-100 *α* and *β*; squamous cell carcinoma for EMA, CD34 and Ki-67; ossifying fibroma for FGF-1 and RUNX-2, normal lung tissue for Sox-9; and inflammatory granulation tissue for MyoD-1. As a negative control, mouse IgG1 (Ki-67, VEGF, CD34, S-100 *β*), IgG2a (EMA, S-100 *α*) and IgG2b (FGF-1), goat IgG (RUNX-2) and rabbit IgG (Sox-9) were used instead of the primary antibodies. Ki-67 immunoreactivity was assessed in areas of the highest staining intensity. At least 500 nuclei were counted in 5 high-power (x400) fields and Ki-67 labeling index was calculated. The number of CD34 positive cells was assessed in 5 medium-power fields. Mean intratumor microvessel density was obtained by calculating the average counts of these 5 fields. These results were analyzed statistically using Welch's test (*P* < .05). The protocol was approved by the Committee on Studies involving Human Beings of Nihon University School of Dentistry at Matsudo (EC 05-002). Informed consent was obtained from patients before surgery.

### 2.3. Clinicopathological Study

Between 1995 and 2007, the files for all cases of oral lipoma were retrieved for study. Clinical data were retrieved from patient records, and all cases were reviewed microscopically and subclassified.

### 2.4. Review of English-Language Literature

The literature from 1960 to 2008 of lipoma with osseous/chondroid differentiation was reviewed.

## 3. Results

### 3.1. Case Report

**Case 1**

**Figure 1:**
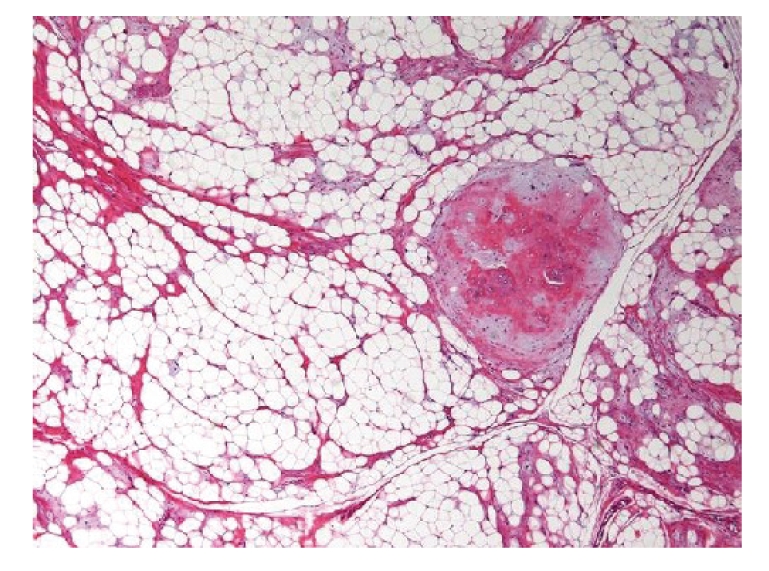
Case 1. Lipoma with chondroid differentiation. Area of mature cartilage nodule is surrounded by normal adipose tissue (HE, original magnification ×40).

A 28-year-old woman presented with a painless relatively hard mass on the dorsal surface in the midline of the tongue that had recently grew slightly. The patient's mother first noticed a tiny nodule when the patient was 6 months old. The lesion had been left because there was no subjective symptom. Examination showed a well-defined hard nodular mass, approximately 16 × 16 × 9 mm in size, which was sharply demarcated within the muscles of the tongue and freely mobile. A yellowish tinge was visible through the overlying mucous membrane and the lesion was firm on palpation. Clinical diagnosis of lipoma with calculus was made and the tumor was completely excised from the tongue under general anesthesia. Gross examination showed a yellowish soft to hard smooth mass measuring 16 × 15 × 12 mm in size. Microscopically, the tumor consisted of a well-circumscribed mass of fatty tissue with a cellular chondroid component. Chondromatous nodules within uniform adipose tissue were seen in large areas of mature fat cells supported by fibrous connective tissue (Figure 1). Pathological diagnosis was made as lipoma with chondroid differentiation.

**Case 2**

**Figure 2:**
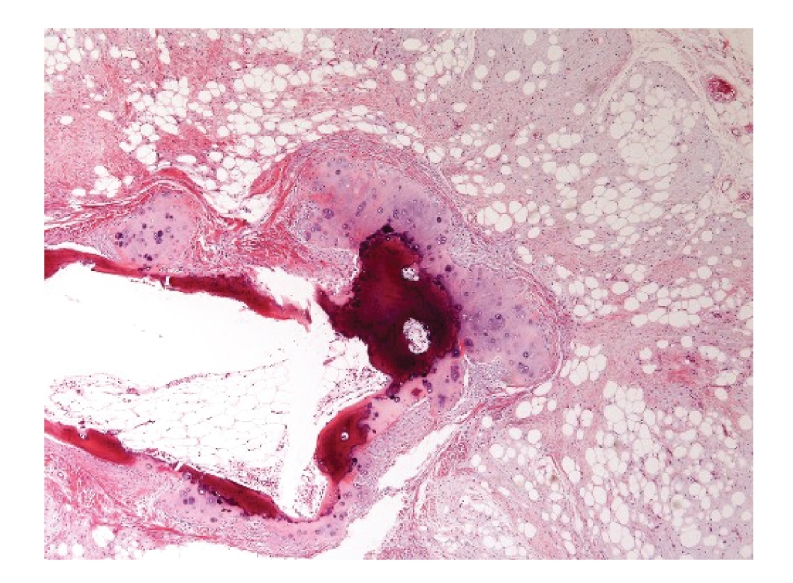
Case 2. Fibrolipoma with osseous/chondroid differentiation. Mature fat cells are supported by fibrous connective tissue with myxoid change and cartilage nodule associated with trabecular bone tissue in it (HE, original magnification ×40).

A 59-year-old man presented with a relatively painless hard mass on the left side of the lower labial vestibule surface, which appeared the sense of incompatibility 2 months prior to consultation. Examination showed a well- defined hard nodular mass, approximately 5 mm in diameter, and freely mobile, and covered with mucosa of normal aspect and color. There was no tenderness, no sign of inflammation. To retrieve more-detailed relativity with the surrounding tissue, CT was taken and showed a lesion in the left side of the lower lip that appeared to be a small mass with areas of little calcification within. This lesion was surgically excised with the clinical diagnosis of fibroma with calcification. Gross examination showed a yellowish soft to hard smooth mass measuring 9 × 5× 5 mm in size. Microscopically, the tumor consisted of a well-circumscribed mass of mature fat cells supported by fibrous connective tissue septa and myxoid tissue characterized by spindle cells. A focal island of consecutive chondroid and woven bone component was surrounded by spindle/fusiform-shaped mesenchymal cells throughout the lesion (Figure 2). Pathological diagnosis was made as fibrolipoma with osseous/chondroid differentiation.

The patients made an uneventful recovery, and after 15 years (Case 1) and 1-year (Case 2) follow-up, there was no sign of recurrence.

### 3.2. Immunohistochemical Study

**Figure 3:**
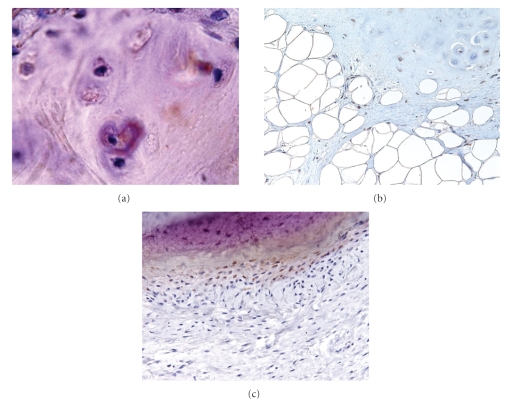
(a) Some chondrocytes reveal positive immunoreactivity for RUNX-2 (RUNX-2, original magnification ×600). (b) Spindle cells around cartilage tissue and some chondroblastic like cells reveal positive immunoreactivity for Sox-9 (Sox-9, original magnification ×200). (c) Osteoblastic cells reveal positive immunoreactivity for RUNX-2 (RUNX-2, original magnification ×200).

Immunohistochemical staining results are shown in Table 1. Most of the adipose cells were stained for S-100 *α* & *β* protein. All chondrocytes were stained for S-100 *α* & *β*, some for RUNX-2 (Figure 3(a)) and some of the outer layer for Sox-9. Spindle cells were divided into 2 groups; peripheral spindle cells around osteo/chondroid tissue and distant spindle cells inside the myxoid area. Peripheral spindle cells around chondroid tissue stained diffusely for S-100 *α* & *β* and Sox-9 (Figure 3(b)), though peripheral spindle cells around osteoid tissue only stained for RUNX-2 (Figure 3(c)). Distant spindle cells inside the myxoid area stained diffusely for S-100 *β* and focally for S-100 *α*, MyoD, Sox-9, and CD34. There was no significant difference between peripheral and distant cells by Ki-67. As for the number of vessels detected by CD34, there were 5.0 vessels in this tumor, and there was no differences among the inside areas. None of the cases stained for either EMA or FGF-1 or with VEGF.

### 3.3. Clinical and Pathological Study

Between 1995 and 2007, the files for all cases of oral lipoma at this department showed 909 cases of nonepithelial tumors in the oral soft tissue. Lipomas represented only 5.0% (*n* = 46) of the cases. Of all the lipoma cases, 27 occurred in males and 19 in females; their mean age was 53.8 years (range: 28–72 years). Most lesions were located in the buccal mucosa (*n* = 16), margin of tongue (*n* = 10), lower lip (*n* = 7), gingiva (*n* = 4), and others. Microscopically, 28 cases (61.0%) were classified as lipoma and 18 (39.0%) as fibrolipoma.

### 3.4. Review of English-Language Literature

The literature from 1960 to 2008 of lipoma with osseous/chondroid differentiation was reviewed. All reported cases are shown in Table 2. Fourteen reports (including 16 cases) were described, and our cases are number 17 and 18. Of all cases, 10 occurred in males and 8 in females; their mean age was 52.4 years (range: 21–81 years). Most lesions were situated in the tongue (*n* = 6), lower lip (*n* = 5) and other areas. Microscopically, 6 cases (33.3%) had an osseous component, 11 cases (61.1%) had a chondroid component, and 1 case (our case, 5.6%) had an osseous/chondroid component. Duration of the lesion varied widely, from 2 months to 30–40 years.

## 4. Discussion

According to our review of the English-language literature,which included our cases, 6 cases were described as lipoma with osseous component [5–7, 9, 14, 15], 11 cases as chondroid component [4, 8–13, 16, 17] and 1 case as fibrolipoma with osseous/chondroid components. Our Case 2 is namely the first report of fibrolipoma with osseous/chondroid differentiation. Osteolipomas are less common than chondrolipomas [18] in the whole body, and almost the same tendency was described in oral lesion. Both of them showed small occurrence. Lipoma with osseous/chondroid differentiaion is extremely rare and occurs mainly in large long-standing lipomas [15, 19]. Case 1 also showed 28-year duration, and this might cause the osseous/chondroid to change into lipoma.

As with many tumors, the etiology of lipomas remains obscure. Chronic irritation, trauma, and spontaneous development have been mentioned. Further indistinct etiology of osseous/chondroid change in lipoma has been discussed and most researchers mentioned that their origin is from different types of undifferentiated mesenchymal cells. Piattelli et al. [14] described two hypotheses of origin of chondroblasts and osteoblasts. The hypothesis is that the neoplastic transformation occurs in multipotential undifferentiated mesenchymal cells that later differentiate into lipoblasts, chondroblasts, or osteoblasts and fibroblasts. Another hypothesis is that only the adipose cells have a neoplastic transformation, and that the cartilage and bone is produced by differentiation of undifferentiated mesenchymal cells of stroma in chondroblasts or osteoblasts.

Immunohistochemical results in this study indicated that there was no proliferative characteristic in mesenchymal (spindle) cells with small positive rates by Ki-67. Chondrocytes and peripheral spindle cells around chondroid tissue with positive findings for S-100 *α*, *β*, and Sox-9 had chondromatous characteristics. Sox-9 belongs to the SOX (Sry-related high-mobility group box) family of transcription factors and is a key regulator of developmental processes including chondrogenesis. RUNX-2 is essential for skeletal mineralization when it stimulates osteoblast differentiation of mesenchymal stem cell and mature chondrocyte differentiation. It also contributes to endothelial cell migration and vascular invasion of developing bones. Positive findings for RUNX-2 in spindle cells around osteoid components and some of chondrocytes indicated osteoblast/chondrocyte characteristics of them. These characteristics are synthesized and thought to be differentiation on account of small positive rate for Ki-67 and negative findings for VEGF and FGF-1 in this study. Osseous/chondroid formation occurred in the central part of these tumors in which a small amount of vessels existed. Multipotential undifferentiated mesenchymal cells had undergone differentiation to osteoblast/chondroblast by topical modification, such as nutrition disorder or asphyxia. The bone formation adjacent to chondroid tissue should not be dystrophic calcification because of its positive findings for RUNX-2 in spindle cells surrounding chondrocytes. These immunohistochemical results supported the conclusion that endochondral ossification and/or perichondral ossification had occurred in these tumor. These results supported lipoma with osseous/chondroid differentiation.

Lipomas are benign mesenchymal neoplasms composed of mature adipocytes and are rare soft tissue tumors with a 2.2% incidence rate of the whole body [20]. A 20% incidence rate of lipoma has been reported in the head and neck region [21]. A 5% incidence rate in oral lesion of this study showed lower than in the head and neck region, but this rate is almost the same tendency compared to previous reports [1, 20, 21]. The average age of the patient of lipoma was 53.8 years old, and male-to-female ratio was 1.4 : 1.0 in this study. Our study supports the current reports that lipomas are generally more common in males than in females (soft tissue), and occurred in adult patients most often between the ages of 40 and 60 years [1, 3]. The predominant locations were the buccal mucosa and the margin of the tongue and the lower lip, where there is plenty of adipose tissue. The patients' characteristics and location distribution, cheek and tongue, were similar to previous reports [1, 20, 22]. Microscopically, 28 cases (61.0%) were classified as lipoma and 18 (39.0%) as fibrolipoma. Tough fibrolipoma is the most frequent subclassification, as Fregnani et al. [1] and Fujimara [4] have reported, but our Case 2 is the first report of fibrolipoma with osseous/chondroid differentiation.

The two cases this reports contributed to pile up of rare lipoma/fibrolipoma with osseous/chondroid differentiation case, and, in addition, immunohistochemical result is contributed to the clarification of the etiology.

## Figures and Tables

**Table 1:** Immunohistochemistry findings of the 2 cases of oral lipoma with osseous/chondroid differentiation.

		S-100 *α*	S-100 *β*	EMA	MyoD1	FGF-1	VEGF	CD34	Ki-67(%)	RUNX-2	Sox-9
Chondrocyte		++	++	−	−	−	−	−	1.3	+ ∼ −	+ ∼ −
Osteocyte		−	−	−	−	−	−	−	1.4	−	−
Adipose cell		+	+	−	−	−	−	−	2.8	−	−
Endothelial cell		−	−	−	−	−	−	5.0 ± 3.6	3.4	−	−
Spindle cell	peripheral*	+	+	−	−	−	−	−	3.2	+	+
		(ch)	(ch)						(ch/os)	(os)	(ch)
	distant**	± ∼ −	+	−	± ∼ −	−	−	± ∼ −	3.3	−	± ∼ −

*peripheral spindle cells around osseous/chondroid tissue

**distant spindle cells inside myxoid area

ch:chondroid

os:osteoid.

**Table 2:** Features of the 18 cases of oral lipoma with osseous/chondroid differentiation described in English literature from 1960 to 2009.

No.	Year	Authors	Age	Sex	Location	Size(cm)	Duration	Component
1	1961	Godby et al. [5]	54	M	Sublingual region	7×6×3	1 year	Osseous
2	1966	Hughes [6]	69	M	Buccal mandibular sulcus	3.5×2.6×1.7	—	Osseous
3	1973	Dutescu et al. [7]	40	M	Left submandibular region	12×6	3 year	Osseous
4	1976	McAndrew and Greenspan [8]	72	M	Lower lip	2.5×1.5	6 mo	Chondroid
5	1982	Allard et al. [9]	81	F	Buccal mandibular sulcus	3.5×2.0	30–40 year	Osseous
			69	F	Lower lip	1×1	2 year	Chondroid
6	1989	Maes and Eulderlink [10]	47	M	Edge of tongue	less than 1	Some mo	Chondroid
7	1992	Fujimura and Enomoto [4]	56	M	Inferior of tongue	1.5×1	2 mo	Chondroid
8	1993	Janne and Franz [11]	21	M	Ramus of left mandible	1.8	—	Chondroid
			43	F	Lower lip	—	1 year	Chondroid
9	1995	Szudrowicz and Jakobi-Róz [12]	52	M	Lower lip	1.7×1.7×1.3	Some mo	Chondroid
10	1997	Hietanen and Makinen [13]	68	F	Dorsal surface of tongue	1.4×1.0	—	Chondroid
11	2001	Piattelli et al. [14]	49	F	Edge of tongue	0.8×0.8	8 year	Osseous
12	2004	Castilho et al. [15]	65	F	Left cheek mucosa	1×1×0.8	—	Osseous
13	2005	Mark [16]	35	M	Lower lip	tiny	2 year	Chondroid
14	2008	Goel et al. [17]	36	F	Edge of tongue	3×2×1	20–30 year	Chondroid
15	2009	Kuyama et al.	28	F	Dorsal surface of tongue	1.6×1.5×1.2	27 year	Chondroid
			59	M	Lower labial vestibule	0.9×0.5×0.5	2 mo	Osseous/chondroid
